# When an antipsychotic working as an antidepressant, do they have a therapeutic window?

**DOI:** 10.1192/j.eurpsy.2025.2076

**Published:** 2025-08-26

**Authors:** I. Hacisalihoglu Aydin, A. Salifu, K. Settle, R. S. El-Mallakh

**Affiliations:** 1 Mood Disorders Research Program, Depression Center, Department of Psychiatry and Behavioral Sciences, University of Louisville School of Medicine; 2 University of Louisville School of Medicine; 3 University of Louisville School of Arts and Sciences, Louisville, United States

## Abstract

**Introduction:**

Therapeutic window in psychopharmacology is the range of drug concentrations that achieve desired effects safely, with treatment failure more likely when levels fall outside this optimal range. The role of dopamine receptor partial agonists (DRPA) in the treatment of depression is a case in point.

**Objectives:**

We discussed the unique mechanisms, and effective antidepressant doses of DRPAs within their therapeutic windows.

**Methods:**

PubMed search was conducted, focusing on randomized controlled trials, open-label studies, and reviews evaluating aripiprazole, brexpiprazole, and cariprazine as augmentation therapies for major depressive disorder in adults.

**Results:**

Clinical trials have investigated aripiprazole, brexpiprazole, and cariprazine as adjuncts to antidepressants, and the effective antidepressant dose is generally lower than the minimal antipsychotic dose. Specifically, the antidepressant doses are 2 – 10 mg for aripiprazole (antipsychotic dose 10 – 30 mg), 2 – 3 mg for brexpiprazole (antipsychotic dose 4 mg), and 1.5 – 3 mg for cariprazine (antipsychotic dose 1.5 – 4.5 mg). This is because at subantipsychotic doses partial agonists increase the dopamine signal, but at antipsychotic doses they reduce the dopamine signal to the level of the intrinsic activity of the drug (generally 25 – 40% of the maximal dopamine signal) which is generally inadequate for an antidepressant response.

**Conclusions:**

DRPAs can increase or reduce the dopamine signal depending on receptor occupancy. At higher receptor occupancy they reduce the dopamine signal, while at lower receptor occupancy (when unoccupied receptors can interact with endogenous dopamine) their intrinsic activity can increase the dopamine signal. Understanding the drug-receptor relationship is crucial, as the assumption that higher doses are always more effective is incorrect.

**Disclosure of Interest:**

None Declared

